# How leisure involvement impacts visitors’ perceived health benefits in urban forest parks: examining the moderating role of place attachment

**DOI:** 10.3389/fpsyg.2024.1493422

**Published:** 2024-11-13

**Authors:** Wei Zhou, Shaozhen Fan, Yuanjing Wu, Guangyu Wang, Siren Lan

**Affiliations:** ^1^College of Landscape Architecture and Art, Fujian Agriculture and Forestry University, Fuzhou, China; ^2^Faculty of Forestry, The University of British Columbia, Vancouver, BC, Canada

**Keywords:** SORM, place attachment, restorative experiences, positive emotions, moderated mediation model

## Abstract

Urban forest park leisure is a significant means for residents to achieve health and well-being, thus possessing high public health value. However, the relationship between visitors’ leisure involvement and perceived health benefits has not been clarified. This study introduced restorative experiences and positive emotions as mediators and place attachment as a psychological moderator, innovatively constructing a stimulus-organism-response-moderator (SORM) integrated model. This moderated mediation model aimed to examine the mechanism through which visitors’ leisure involvement influenced perceived health benefits. A field survey was conducted in Fuzhou National Forest Park in Fujian, China, resulting in the collection of 588 valid questionnaires. The results showed that visitors’ leisure involvement positively impacted restorative experiences and positive emotions. Restorative experiences and positive emotions completely mediated the indirect relationship between visitors’ leisure involvement and perceived health benefits. Place attachment enhanced the impact of restorative experiences on perceived health benefits, thereby positively moderating the mediation effect of restorative experiences. Place attachment also diminished the impact of positive emotions on perceived health benefits, thereby negatively moderating the mediation effect of positive emotions. Therefore, significant differences existed in the psychological processes involved in acquiring perceived health benefits among visitors with different levels of place attachment. Our findings might enrich the existing knowledge of place attachment and forest health benefits, providing valuable references for designing and optimizing urban forest parks.

## 1 Introduction

The intensification of urbanization has led to the reduction in natural environments and the transformation of human settlements, thereby resulting in significant lifestyle and ecological changes (Clement, 2010). This shift from natural to urban settings has led to the lack of human exposure to nature and a subsequent increase in the occurrence of psychological disorders such as depression and insomnia (Berry et al., 2020; McDougall et al., 2021; Liu et al., 2022). In the post-pandemic era, people have started rethinking about the meaning of leisure and its value in their lives (Cui et al., 2022; Iso-Ahola and Baumeister, 2023). Pursuing rehabilitation and health has prominently motivated residents to engage in outdoor leisure activities, such as jogging, hiking, mountaineering, and so forth (Pietilä et al., 2015; Morse et al., 2021). Compared with the urban environment, natural settings are closely related to people’s physical and mental health (Yu et al., 2018; Li et al., 2023). Buckley et al. (2019) indicated that natural protected areas worldwide generate approximately $6 trillion in mental health value annually. As an integral part of urban green space, urban forest parks have abundant natural resources and stronger interactions with residents (Chen B. et al., 2018; Lin et al., 2023). Research has shown that urban forest parks, by offering rich natural experiences, can significantly reduce stress and mental fatigue, thereby improving psychological well-being (Sonntag-Öström et al., 2015; Vujcic and Tomicevic-Dubljevic, 2018). Additionally, forest environments can provide tangible physiological health benefits by enhancing cardiovascular function and boosting immune system performance (Park et al., 2021; Tsao et al., 2022). Therefore, visitors prefer urban forest parks with markedly restorative characteristics as optimal leisure spots for self-healing.

Participation in leisure activities is referred to as “leisure involvement” in the field of leisure studies (Havitz and Dimanche, 1990). This interdisciplinary field explores how individuals engage in leisure activities and examines the meaning and benefits these activities bring to participants. Specifically, leisure involvement refers to the psychological energy and attention that individuals invest in leisure activities (Li et al., 2020). This investment not only enhances emotional connections but also brings significant psychological health benefits (Geng et al., 2023). Research has shown that high levels of leisure involvement can significantly reduce stress, increase well-being, and improve life satisfaction (Chun et al., 2023; Geng et al., 2023). However, the after-effects of leisure involvement, particularly the psychological pathways related to health benefits, remain underexamined. Moreover, empirical research focusing on urban forest parks as leisure destinations is lacking. In recent years, the focus of urban forest landscape planning has gradually shifted from economic and ecological benefits to public health services (Lan et al., 2023). Therefore, exploring the influence mechanism between leisure involvement and visitors’ perceived health benefits in urban forest parks is crucial for developing strategies to enhance leisure quality and inform effective forest landscape management.

The stimulus-organism-response (SOR) theory, a key framework in environmental psychology, posits that the individual’s psychological states are the key mediating variables influencing their behavioral response when exposed to external environmental stimuli (Mehrabian and Russell, 1974). However, many studies have considered psychological states only as part of leisure benefits, ignoring the contribution of different types of psychological states to visitors’ perceived health benefits. Meanwhile, some scholars have pointed out that forest environments can help visitors achieve two positive psychological states: restorative experiences and positive emotions (Liu et al., 2020; Wu et al., 2023; Yan et al., 2024). Prolonged exposure to forests significantly enhances the feeling of vitality and recovery (Takayama et al., 2014), while reducing negative emotions such as anxiety, depression, tension, fatigue, and confusion (Muro et al., 2022). Additionally, individuals experience positive emotions, which subsequently lead to changes in cognition and behavior. This process can be described as a pathway of stimulation, reception, purification, insight, and restoration, ultimately resulting in improved health outcomes (Oh et al., 2020). Further research has suggested that restorative experiences following forest leisure directly improve mental health by reducing stress and cognitive fatigue (Hartig et al., 1991; Chen H.-T. et al., 2018), whereas enjoyable leisure activities enhance positive emotions, boosting emotional resilience and overall life satisfaction (Chen et al., 2022). These two psychological states are key predictor variables of visitors’ perceived health benefits and are closely related to the strength of the person-place connection (Liu et al., 2020; Guo et al., 2024).

Humans have an inherent affinity for nature, particularly open, low-risk environments (Kaplan, 1995). This predisposition underlies people’s emotional connections with natural places, influencing their leisure choices and experiences (Heidari et al., 2021). As one of the key concepts of person–place emotions; place attachment provides an important research direction for explaining the relationship between psychological states of leisure and perceived health benefits (Williams and Vaske, 2003). Previous research has validated the positive role of place attachment in moderating visitors’ behavioral intentions (Kim et al., 2017), but this insight has not yet received adequate attention in leisure research. Place attachment implies a deep emotional bond between an individual and a specific environment that enhances the satisfaction derived from leisure activities (Lee et al., 2012), potentially amplifying the associated health benefits. Therefore, place attachment may moderate the relationship between visitors’ leisure involvement and perceived health benefits. In this study, we incorporate place attachment as a psychological moderator into the SOR theoretical framework, thereby constructing a stimulus-organism-response-moderator (SORM) integrated model.

Using the SORM integration model as a framework, this study explored three primary objectives: (1) to examine the relationships among leisure involvement, restorative experiences, positive emotions, and perceived health benefits in the context of urban forest parks; (2) to reveal the mediating role of restorative experiences and positive emotions, thereby illustrating how these psychological states translate leisure activities into measurable health benefits; and (3) to examine whether and how place attachment moderates these mediating effects on the influence of leisure involvement on perceived health benefits. The insights gained from this study offer valuable directives for the design and management of urban forest parks to maximize health benefits for visitors, emphasizing the importance of tailoring experiences to different levels of place attachment. We encourage urban planners and park developers to integrate these insights into their landscape management strategies, ensuring that urban forest parks are not only leisure spaces but also vital health resources for cities.

## 2 Literature review and research hypotheses

### 2.1 Stimulus-organism-response-moderator (SORM) integrated model

The stimulus-organism-response (SOR) theory, introduced by Mehrabian and Russell (1974), offers a theoretical framework for studying user behavior. The stimulus (S) represents external environment or events that impact the organism (O) by driving cognitive and emotional processes, ultimately manifesting in specific behavioral patterns or psychological responses (R) (Wang et al., 2022). Moreover, some scholars suggested that external stimuli can directly drive corresponding behavioral responses (Dong et al., 2022). The SOR theory emphasizes the mediating role of an individual’s internal state, which encompasses a wide range of psychological dimensions, including satisfaction, environmental commitment, and destination attachment (Zhu et al., 2020; Tang et al., 2022; Guleria et al., 2023). This theory effectively captures the complex psychological processes underlying behavioral changes, making it applicable to various research domains, such as consumer decision-making, subjective well-being, and the pro-environmental behaviors exhibited by visitors (Zhu et al., 2020; Wang et al., 2022; Zhou et al., 2023). This wide-ranging applicability of the SOR framework not only highlights its utility in understanding the dynamic interactions between environmental stimuli and individual responses but also reflects its extensibility. For instance, Huang (2023) integrated the SOR framework with the technology acceptance model to explore smartphone use behavior among older adults.

Furthermore, scholars across various fields have recognized that the existing SOR theory overlooks the boundary conditions that influence the mechanisms driving individual behavioral decisions and psychological responses. Consequently, there has been an effort to integrate moderating variables into the SOR framework (Dong et al., 2022; Duong and Nguyen, 2024). This shift aligns with broader developments in environmental psychology, where there is a growing emphasis on exploring individual psychological moderators within explanatory models. For instance, Zhou et al. (2023) explored how eco-centrism moderated the relationship between restorative perception and pro-environmental behaviors among forest park visitors. Similarly, Dong and Qu (2023) investigated the moderating role of travel memory in the effects of subjective well-being and self-congruity on destination attachment. Although existing research has attempted to introduce moderators into the SOR framework, there has not yet been a systematic effort to adjust the framework structure. This study addresses this gap by integrating psychological moderators into the SOR framework, proposing a stimulus-organism-response-moderator (SORM) integrated model to further refine and expand the theoretical framework.

This integrated model is proposed for several key reasons. First, incorporating psychological moderators can significantly enhance the explanatory and predictive power of the SOR framework, allowing researchers to more accurately capture individual behavioral changes across different contexts and uncover the complex interaction mechanisms between environmental stimuli and individual behavior. Additionally, psychological moderators can support the development of personalized and context-specific intervention strategies, making research findings more applicable in practice by addressing the needs of different individuals or groups, thereby increasing the practical value of the research. Furthermore, the inclusion of psychological moderators fosters the expansion and innovation of the SOR framework, providing fresh perspectives for future research.

#### 2.1.1 Stimulus: leisure involvement

The concept of ego involvement was initially introduced by Sherif and Cantril (1947). By the 1980s, this concept was expanded to the field of leisure studies, introducing the idea of leisure involvement (Selin and Howard, 1988). As a broad social behavior, leisure activities refer to non-work activities that individuals engage in during their free time (Iso-Ahola and Baumeister, 2023). Leisure involvement essentially reflects an individual’s attitude toward these activities, indicating the strength of the cognitive connection between the individual and the stimulus object (Geng et al., 2023). Subsequently, this concept has significantly shaped leisure studies, with numerous scholars exploring the meaning and structure of leisure involvement. For example, Mclntyre (1989) defined leisure involvement as the internal psychological process of an individual’s participation in leisure activities, which can be represented by dimensions such as attraction, self-expression, and centrality. Similarly, Wiley et al. (2000) described it as the level of meaning and interest a leisure activity imparted to individuals, influencing their leisure decisions. Additionally, some researchers have studied leisure involvement as a unidimensional concept (Chen et al., 2013). In this study, we defined leisure involvement as a perceptual state tied to forest leisure activities, encompassing the individual’s perceived leisure pleasure and self-identity, thus reflecting the activity’s attractiveness and importance.

Traditionally, stimulus refers to environmental characteristics or external cues that influence an individual’s psychological or cognitive state (Mehrabian and Russell, 1974; Huang, 2023). In contrast, leisure involvement is typically regarded as a subjective, internal psychological experience, reflecting the degree of an individual’s engagement in leisure activities (Zaichkowsky, 1985; Selin and Howard, 1988). From this perspective, leisure involvement appears to align more closely with the organism (O) rather than the stimulus (S) category. However, as research has advanced, it has become increasingly recognized that any factor capable of triggering psychological or behavioral responses, whether external or internal, can be considered a stimulus (Dong et al., 2022; Huang, 2023; Duong and Nguyen, 2024). Therefore, while leisure involvement is usually viewed as a psychological variable, it can be redefined as a stimulus within the context of the SORM model. This is because leisure involvement reflects an individual’s psychological connection to leisure activities, and this connection itself can serve as a precursor to psychological responses (Li et al., 2020; Geng et al., 2023). Thus, leisure involvement represents an intrinsic motivational force within leisure contexts, playing a role similar to that of traditional external environmental factors in eliciting and sustaining cognitive and emotional responses.

#### 2.1.2 Organism: restorative experiences and positive emotions

Through positive interactions with the environment, individuals can achieve restorative experiences, that is, recovering from negative states associated with psychological fatigue and stress (Kaplan, 1995; Wu et al., 2023). Natural environments are widely recognized for their exceptional restorative potential (Hartig et al., 1991; van den Berg et al., 2003). Additionally, some human-designed natural environments, such as urban parks, green streets, and urban greenways, can also provide similar restorative experiences (Gao et al., 2019; Ojala et al., 2019; Li et al., 2023). To explain the restorative properties of natural environments, Kaplan (1995) proposed the attention restoration theory (ART), which emphasizes the role of external environments in restoring cognitive functions, particularly attention. According to this theory, the human preference for natural environments is an instinctive response rooted in evolutionary genetics, where natural exposures stimulate indirect attention, thereby preventing the overuse of direct attention. Based on ART, Korpela et al. (2008) developed the restoration outcome scale, which focuses on measuring visitors’ psychological restoration experiences and has been recognized by many scholars (Ojala et al., 2019; Menzel et al., 2024). In this study, “restorative experience” was conceptualized as the alleviation of energy depletion in visitors, leading to various positive changes such as stress reduction and enhanced vitality.

Moreover, intrinsic motivation often drives individuals to engage in specific leisure activities to fulfill their genuine needs, thereby fostering the emergence of positive emotions (Liang and Peng, 2018; Iso-Ahola and Baumeister, 2023). Positive emotions are a psychological state that arises when leisure needs are satisfied in response to particular environmental stimuli (Kong et al., 2022). According to Fredrickson (2001), positive emotions are unique, immediate responses to valued experiences, including joy, desire, contentment, interest, peace, love, and motivational incentives. The stress recovery theory suggests that most people prefer interacting with nature and such interactions can transform stress into positive emotions and prevent negative thoughts (Ulrich et al., 1991; van den Berg et al., 2003). Additionally, positive emotions can broaden cognitive scope, enhance behavioral performance, and contribute to the development of physical and psychological resources (Fredrickson, 2001; Lin et al., 2023).

Existing research widely recognizes leisure involvement as a key antecedent variable and employs diverse methods to explore its after-effects. Despite this extensive coverage, a notable paucity of evidence specifically addressing the perceived health benefits associated with visitors’ leisure activities exists. This gap underscores the need to investigate further the psychological processes underpinning visitors’ perceived health benefits, particularly focusing on restorative experiences and positive emotions. Therefore, in the SORM model, the “Organism” component includes restorative experiences and positive emotions, representing the individual’s internal state.

#### 2.1.3 Response: perceived health benefits

Modern health is conceptualized not merely as the absence of illness but as a state of positive physical, psychological, and social adjustment (Leonardi, 2018). Environmental psychologists have asserted that the natural environment is favored for its inherent aesthetic and self-healing properties, offering extensive health benefits (Kim et al., 2014; Oh et al., 2020; Muro et al., 2022). These benefits can be broadly divided into three categories: short-term recovery from psychological stress and negative emotions, reduction in physical illnesses, and long-term enhancement of social well-being (Kim et al., 2014). This study defined perceived health benefits as the improvements in psychological, physical, and social performances that individuals gain through urban forest park leisure. The assessment of health benefits incorporates various metrics, including physiological data such as electromyography values and heart rate variability (Park et al., 2021; Tsao et al., 2022), affective tests, self-reported emotional states, and self-rated health (Velarde et al., 2007; Pietilä et al., 2015). Notably, using questionnaires to evaluate visitors’ perceived health benefits offers a rapid and effective method to measure these benefits within leisure destinations. This approach has been validated in previous studies, confirming its utility in examining the relationship between environmental stimuli and health benefits (Peschardt and Stigsdotter, 2013). As argued by Guo et al. (2024), health benefits are an important spatial attribute of urban green spaces, serving as a key outcome variable in human psychological responses. Therefore, the perceived health benefits can be considered the “Response” variable in the SORM model.

#### 2.1.4 Moderator: place attachment

Attachment theory posits that humans possess a biological instinct to seek proximity and establish emotional bonds with attachment figures, which is fundamental for survival (Raymond et al., 2010). Williams and Vaske (2003) proposed the concept of place attachment, defining it as a multidimensional structure that included both material dependencies and emotional connections rooted in an individual’s cognition, feelings, and behaviors. This structure comprised place dependence—the functional attachment to the material resources of a place—and place identity, which reflected the emotional value and symbolic meaning assigned to a place (Williams and Vaske, 2003; Dong and Qu, 2023). Leisure environments and activities often hold unique feelings and memories for visitors, which imbue these places with distinctive meanings over time, fostering familiarity and a strong sense of belonging (Ganji et al., 2021; Tao et al., 2022). This process can evoke deep emotional attachment to urban forest parks, indirectly enhancing or diminishing the responsiveness of psychological states, thereby moderating the perceived health benefits. In this context, place attachment can be regarded as a psychological moderator. For instance, when an individual has a strong attachment to a particular leisure environment, this attachment amplifies their sensitivity to other stimuli within the environment, making them more likely to perceive the restorative or emotionally uplifting qualities of the setting (Liu et al., 2020). This amplifying effect positions place attachment as a dynamic and contextual moderator within the SORM model.

### 2.2 Research hypotheses

#### 2.2.1 Leisure involvement and perceived health benefits

Individuals often replenish their energy through leisure activities when faced with the demands of excessive life and work stress (Iso-Ahola and Baumeister, 2023). Leisure activities, defined as voluntary, creative, and recreational behaviors undertaken during free time, offer numerous health benefits that are typically influenced by the level of psychological involvement in the activities (Chun et al., 2023; Geng et al., 2023). Research has suggested that leisure involvement encompasses the process of receiving environmental stimuli at a leisure destination, which affects the continuity and desire for leisure experiences and triggers various health benefits (Geng et al., 2023). For instance, Tao et al. (2022) found that higher levels of leisure involvement and stronger flow experiences enhanced residents’ attachment and leisure benefits, ultimately improving their well-being and quality of life. Similarly, Lee et al. (2022) highlighted the critical role of cultural and leisure services provided by forest ecosystems in promoting residents’ health, underscoring their significant value to urban forests. Furthermore, Kim et al. (2011) identified a positive correlation between serious leisure involvement, life satisfaction, and perceived health. Building upon these insights, this study proposed the following hypothesis:

*Hypothesis 1 (H1):* Leisure involvement has a significant positive effect on perceived health benefits.

#### 2.2.2 The mediating role of restorative experiences and positive emotions

Previous studies indicated that leisure activities not only provided a mental respite from daily stressors but also contributed to the sustained replenishment of physical and mental energy—key factors in facilitating healthy recovery (Cho and Park, 2018; Chun et al., 2023). In their preferred leisure environments, people are more inclined to engage in leisure activities with greater enthusiasm and improved behavioral performance, making it easier to evoke positive restorative experiences (Kaplan, 1995). Moreover, the extent of involvement in leisure activities is correlated with varying levels of positive emotions, such as delight, passion, and satisfaction (Liang and Peng, 2018; Li et al., 2020). Compared to those who engage in leisure activities sporadically, individuals who participate regularly report substantially greater psychological benefits and well-being (Chen et al., 2022). Two months of deep involvement in forest therapy can improve mood and reduce anxiety in individuals with affective, such as depression or psychotic disorders (Bielinis et al., 2020). These positive effects can last for up to 3 months (Sonntag-Öström et al., 2015). Kim et al. (2014) highlighted that leisure activities enabled older adults to better manage negative emotions and enhance their positive emotional experiences.

The conservation of resources theory further supports this by illustrating how transformations between different types of physical and mental resources occur, suggesting that acquiring one type of resource can foster the development of others (Hobfoll, 1989). For example, Cho and Park (2018) found that achieving psychological detachment from work during leisure activities led to short-term mental health recovery and a reduction in negative affective states. Similarly, Kim et al. (2016) observed that sustained physical and cognitive engagements in leisure activities among older adults helped maintain their physical vigor and yielded psychological benefits. Liang and Peng (2018) demonstrated that a good fit between the recreationist and the environment significantly enhanced positive emotional responses, thus promoting physical and mental relaxation. Given these insights, the restorative experiences and positive emotions gained during the process of leisure involvement are likely key predictors of perceived health benefits. Based on these findings, this study advanced the following hypotheses:

*Hypothesis 2 (H2):* Leisure involvement has a significant positive effect on restorative experiences.

*Hypothesis 3 (H3):* Restorative experiences have a significant positive effect on perceived health benefits.

*Hypothesis 4 (H4):* Leisure involvement indirectly affects perceived health benefits through the mediating effect of restorative experiences.

*Hypothesis 5 (H5):* Leisure involvement has a significant positive effect on positive emotions.

*Hypothesis 6 (H6):* Positive emotions have a significant positive effect on perceived health benefits.

*Hypothesis 7 (H7):* Leisure involvement indirectly affects perceived health benefits through the mediating effect of positive emotions.

#### 2.2.3 The moderating role of place attachment

Place attachment fulfills both the material and spiritual needs of individuals, reducing anxiety and stimulating positive behavioral and psychological responses (Guo et al., 2024). Environmental psychologists suggest that individuals with high levels of place attachment are more likely to engage deeply in leisure destinations (Lee et al., 2012), thus facilitating the translation of psychological benefits, such as restorative experiences and positive emotions, into perceived health benefits. Moreover, place attachment influences how visitors process restorative information in natural environments, with those highly attached more likely to perceive and transform restorative experiences into health benefits, unlike those with lower levels of attachment (Liu et al., 2020). Therefore, place attachment may moderate the process of perceived health benefits for visitors. Given these insights, this study proposed the following hypotheses:

*Hypothesis 8 (H8):* Place attachment enhances the impact of restorative experiences on perceived health benefits, significantly and positively moderates the mediating effect of restorative experiences.

*Hypothesis 9 (H9):* Place attachment enhances the impact of positive emotions on perceived health benefits, significantly and positively moderates the mediating effect of positive emotions.

### 2.3 Conceptual framework

**Figure 1:**
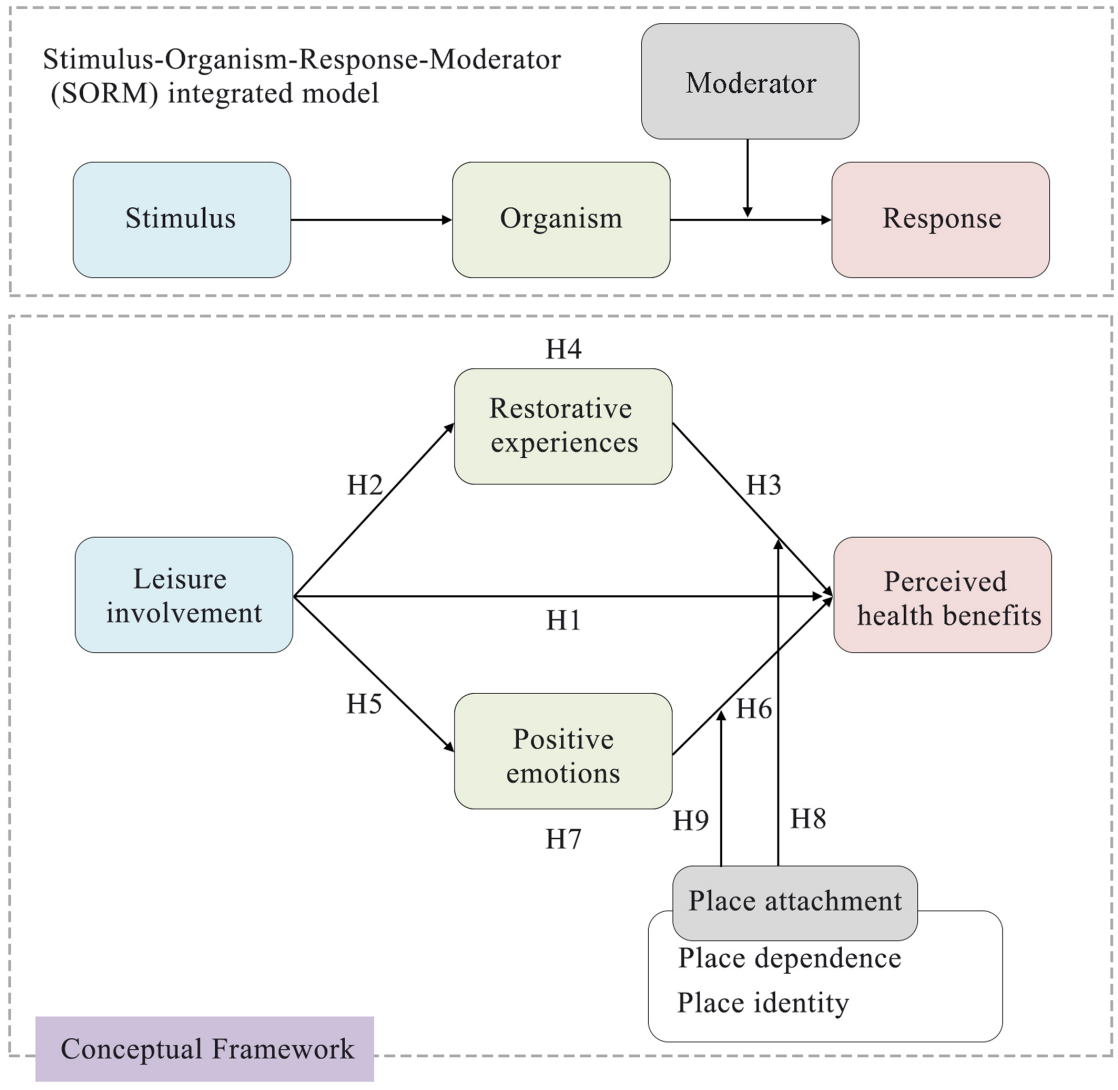
Conceptual framework of the study.

Based on the SORM integrated model, this study developed a conceptual framework containing hypotheses, as shown in Figure 1. Specifically, the study hypothesized that the pathway from environmental stimuli (leisure involvement) to psychological response outcomes (perceived health benefits) operated through the psychological state of the organism, encompassing restorative experiences and positive emotions. Additionally, place attachment was introduced as a moderating variable. Through this framework, the study not only validated the effectiveness of the SORM integrated model in explaining visitors’ perceived health benefits but also provided a more detailed perspective on how interactions between the environment and psychological factors shape health outcomes.

## 3 Materials and methods

### 3.1 Study site

**Figure 2:**
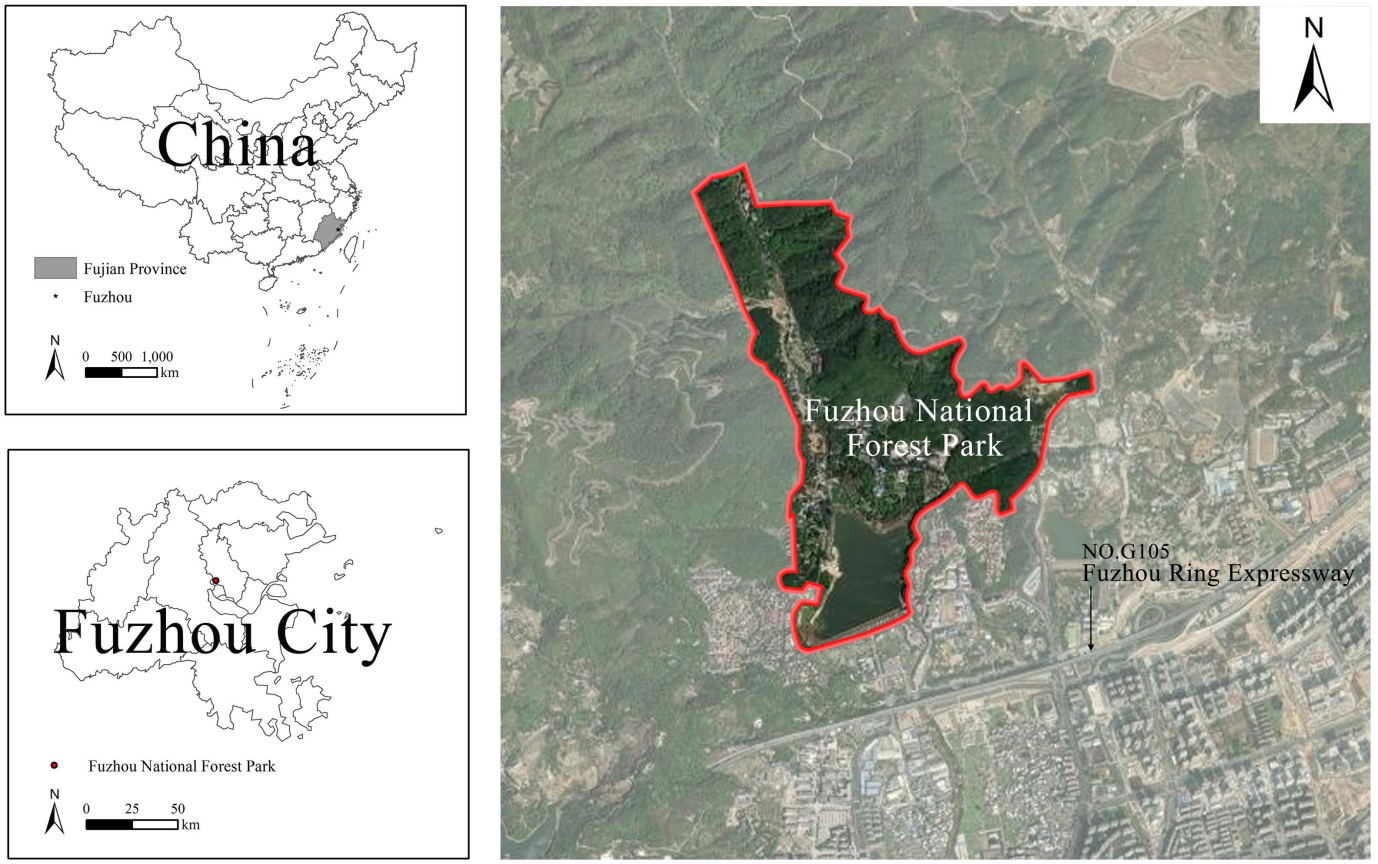
Location map of Fuzhou National Forest Park.

The study was conducted at Fuzhou National Forest Park, located in the northern mountainous part of Fuzhou City, China (Figure 2). Designated as a national 4A-level scenic spot in 2000, this site holds the distinction of being the first national urban forest park in Fujian Province that combines scientific research, recreation, sightseeing tours, and other functions. Spanning approximately 860 hectares, the park boasts lush natural settings and a unique forest microclimate, earning it the moniker “the lungs of Fuzhou.” The rich forests, abundant water bodies, and diverse flora and fauna of the park not only satisfy visitors’ desires to connect with nature but also make it a popular destination for leisure and wellness activities.

### 3.2 Questionnaire design

This study used a two-part questionnaire to collect empirical data. The first part gathered basic personal information from respondents, including gender, age, educational background, occupation, monthly income, frequency of visits, and partner. The second part, which formed the core of the questionnaire, comprised scales to measure the five key variables of the study. These scales and their items have been previously validated and widely used in relevant literature.

First, leisure involvement was assessed using a 5-question scale adapted from Havitz and Dimanche (1990) and Tao et al. (2022). The selected questions capture visitors’ personal enjoyment, time and resource investment, and the significance of leisure activities within the context of urban forest parks. Questions more relevant to competitive or specialized leisure settings were omitted to maintain the relevance and applicability of the scale to general leisure experiences in a natural setting. The Cronbach’s alpha value of the leisure involvement scale was 0.923, indicating high reliability.

Second, restorative experiences were measured using 5 questions from Korpela et al. (2008) and Ojala et al. (2019). The selected questions focus on the psychological restoration and mental recovery that visitors experience in the forest environment, such as stress relief and enhanced alertness. The Cronbach’s alpha value of this scale was 0.942, indicating it is a reliable measurement.

Third, positive emotions were evaluated using 4 questions based on Watson et al. (1988) and Moon (2020). The selected questions capture visitors’ immediate emotional responses to their leisure experience, such as feelings of fulfillment and the alleviation of negative emotions. Questions focusing on longer-term emotional states or irrelevant affective responses were omitted to ensure the scale measured short-term emotional impacts in the forest environment. The Cronbach’s alpha value was 0.910, indicating reliable measurement.

Fourth, place attachment, encompassing the dimensions of place dependence and place identity, was measured using a 7-question scale from Williams and Vaske (2003) and Dong and Qu (2023). The questions were selected to assess both the functional (place dependence) and emotional (place identity) connections that visitors form with the urban forest park. The Cronbach’s alpha value of this scale was 0.931, indicating high reliability.

Finally, perceived health benefits were assessed by five statements from Peschardt and Stigsdotter (2013) and Guo et al. (2024). The selected questions address cognitive, physical, and social health improvements, which are particularly relevant in natural environments like urban forest parks. The Cronbach’s alpha value of this scale was 0.900, indicating it is a reliable measurement.

The questions were carefully modified to ensure that each scale reflected the unique features and experiences of the urban forest park setting, enhancing both the relevance and the readability of the questionnaire. All items were measured on a 7-point Likert scale, where 1 represented “strongly disagree,” and 7 represented “strongly agree.”

### 3.3 Sampling and data collection

The research team conducted field surveys at Fuzhou National Forest Park from September 26 to October 6, 2023 (7:00 am to 11:00 am and 14:00 to 17:00). Due to the primary objective of this study being to understand the experiences and perceived health benefits of visitors actively engaging in leisure activities within urban forest parks, the convenience sampling method was employed. This method was chosen for its practicality in accessing a specific population—visitors who were already present in the forest park and willing to participate in the study. This approach ensured that the sample accurately reflected the characteristics and perceptions of those who actively choose to visit urban forest parks for leisure, which aligns with the focus of our research. While convenience sampling may limit the generalizability of the findings, it provides a dataset for exploring the targeted visitor group.

Before participating, visitors were given a brief overview of the study’s purpose and asked for their consent to participate. Only visitors who had been in the park for at least 1 h were invited to fill out the questionnaire. This selection criterion ensured that respondents possessed an adequate understanding of the park’s leisure environment and a certain level of place attachment, thereby enhancing the validity and reliability of the collected data. After discarding incomplete or inconsistently filled questionnaires, a total of 588 valid responses were collected, resulting in a valid response rate of 84%.

### 3.4 Statistical analysis

The reliability and validity of the scales were evaluated using SPSS 23.0 and AMOS 23.0 software. Structural equation modeling (SEM) was subsequently conducted with AMOS 23.0 to assess the relationships among variables and validate the research hypotheses. Additionally, the moderated mediation effect was examined using PROCESS macro in SPSS 23.0.

Harman’s single-factor test was performed before initiating the main analyses to address the potential for common method bias (CMB) inherent in questionnaire-based data collection. The results revealed that the first unrotated factor explained 38.08% of the overall variance, which was below the threshold of 50% often cited for significant CMB (Podsakoff and Organ, 1986). This indicated that CMB posed no significant concern in our study data.

## 4 Results

### 4.1 Profile of the respondents

As shown in Table 1, the sample demographics revealed that 52.7% of respondents were women. Age distribution was mainly between 21 to 35 and 36 to 50 years old, accounting for 43.9 and 34.4% of the sample, respectively. Further, 65% of respondents had attended university or had a bachelor’s degree. The primary occupational groups were corporate employees and student, accounting for 27.6 and 27.0% of the sample, respectively. Income-wise, more than half of the respondents (53.8%) reported monthly earnings between Chinese Yuan (CNY) 3,001 and CNY 10,000. The majority visited the park with family (53.9%) or friends (29.3%). Regarding repeat visits, 55.1% of respondents indicated they were on their sixth to tenth visit to the park. Overall, the sample displayed a reasonable age structure and a high level of education. In addition, it was common for respondents to visit the park multiple times, enhancing the validity of place attachment assessments. Therefore, the sample was representative.

**Table 1:** Respondent information (*N* = 588).

Variable	Category	Sample	Percentage (%)	Variable	Category	Sample	Percentage (%)
Gender	Male	278	47.3	Age (year)	20 and below	97	16.5
	Female	310	52.7		21–35	258	43.9
Education	Junior high school or below	28	4.8		36–50	202	34.4
	High school or equivalent	142	24.1		51–65	26	4.4
	Undergraduate	382	65.0		66 and above	5	0.9
	Graduate and above	36	6.1	Partner	Alone	54	9.2
Occupation	Corporate employees	162	27.6		Family	317	53.9
	Private business owner	62	10.5		Friends	172	29.3
	Student	159	27.0		Boyfriend or girlfriend	39	6.6
	Government worker	111	18.9		Other	6	1.0
	Other	94	16.0	Number of visits	First	104	17.7
Monthly income	Under CNY 3000	159	27.0		2–5	120	20.4
	CNY 3001–6,000	205	34.9		6–10	324	55.1
	CNY 6001–10,000	111	18.9		11 and above	40	6.8
	Over CNY 10,001	113	19.2				

### 4.2 Measurement model testing

The skewness and kurtosis absolute values of the scale’s individual items ranged from 0.031 to 0.747 and 0.055 to 0.618, respectively, suggested that the data were approximately normally distributed. This near-normal distribution was conducive to employing covariance-based SEM (CB-SEM) for more detailed analysis (Dash and Paul, 2021). The results of descriptive statistics, reliability, and validity tests for each variable are shown in Tables 2, 3, respectively.

**Table 2:** Results of confirmatory factor analysis for the measurement model.

Variable	Code	Item	Standardized factor loading	*t-*value
Leisure involvement	LI1	I get pleasure during my leisure activities here.	0.841	Fixed
	LI2	I am willing to spend time/money on leisure here.	0.826	24.448
	LI3	Leisure activities here are very important to my life.	0.834	24.972
	LI4	I am glad to let others know that I’m relaxing here.	0.845	25.114
	LI5	I am willing to share my leisure experiences with family/friends.	0.859	25.903
Restorative experiences	RE1	The leisure experience here always leaves me feeling relaxed and restored.	0.908	Fixed
	RE2	I feel calmer after spending time in the forest park.	0.897	30.425
	RE3	Here, I can forget the worries of daily life.	0.809	25.279
	RE4	Here, my energy and alertness clearly increase.	0.895	30.479
	RE5	The leisure experience here helps to clear and clarify my thoughts.	0.864	31.279
Positive emotions	PE1	The leisure experience here makes me feel content and fulfilled.	0.871	Fixed
	PE2	Here, I can gain new enthusiasm and energy to handle daily tasks.	0.838	25.803
	PE3	Here, my negative emotions are alleviated.	0.808	24.416
	PE4	I was satisfied with my leisure experience here.	0.874	27.561
Place dependence	PD1	The leisure experience here is unparalleled and cannot be replicated by any other parks.	0.846	Fixed
	PD2	My leisure experience here is more fulfilling than in other parks.	0.841	25.319
	PD3	Compared to other parks, this park is more conducive to engaging in various leisure activities.	0.870	26.676
	PD4	Compared to other parks, I prefer the natural environment here.	0.874	27.078
Place identity	PI1	If the situation allows, I will take the initiative to extend my leisure time here.	0.883	Fixed
	PI2	I am very attached to this forest park.	0.844	25.960
	PI3	I identify strongly with leisure environment of this forest park.	0.830	25.443
Perceived health benefits	PH1	Here, my concentration is enhanced	0.812	Fixed
	PH2	Here, my fatigue is reduced.	0.800	21.596
	PH3	Here, my physical vitality is recovered.	0.813	21.916
	PH4	Here, my social performance is enhanced.	0.749	19.755
	PH5	Here, my overall health improved.	0.835	22.837

**Table 3:** Mean, standard deviation, composite reliability, average variance extracted, Cronbach’s alpha, and correlations (Pearson’s *r*) between variables.

	Mean	SD	AVE	CR	Cronbach’s alpha	1	2	3	4	5	6
1. Leisure involvement	5.093	0.887	0.707	0.927	0.923	1					
2. Restorative experiences	4.828	0.936	0.766	0.943	0.942	0.414	1				
3. Positive emotions	4.954	0.849	0.719	0.911	0.910	0.318	0.150	1			
4. Place dependence	5.007	0.917	0.736	0.918	0.917	0.348	0.597	0.076	1		
5. Place identity	4.957	0.911	0.727	0.889	0.888	0.268	0.538	0.077	0.747	1	
6. Perceived health benefits	5.408	0.729	0.644	0.900	0.899	0.314	0.394	0.444	0.392	0.455	1
Squared root of average variance extracted						0.846	0.875	0.848	0.858	0.852	0.802

SD, standard deviation; AVE, average variance extracted; CR, composite reliability.

The results of the reliability test showed that Cronbach’s alpha values for all variables were between 0.888 and 0.942, which exceeded the critical standard. Also, the composite reliability (CR) values were all above 0.8, indicating that the data had sufficient internal consistency. The CB-SEM program, AMOS 23.0, was used to conduct confirmatory factor analysis. The results showed that the standardized factor loadings of each item were between 0.749 and 0.907, which exceeded 0.7 and were significant, with no need to eliminate the factors. The average variance extracted (AVE) for all measurement models exceeded 0.6, demonstrating convergent validity. The discriminant validity test results of the measurement model indicated that the square root of the AVE for each variable was higher than the Pearson correlation coefficient between that variable and all others. In addition, the heterotrait–monotrait ratios ranged from 0.084 to 0.827 (Table 4), well below the critical standard of 0.85 (Henseler et al., 2015), further validating the discriminant validity of the measurement models.

**Table 4:** Heterotrait–monotrait results.

Variable	1	2	3	4	5
1. Leisure involvement	–				
2. Restorative experiences	0.445	–			
3. Positive emotions	0.347	0.161	–		
4. Place dependence	0.378	0.643	0.084	–	
5. Place identity	0.296	0.590	0.085	0.827	–
6. Perceived health benefits	0.345	0.428	0.491	0.431	0.509

### 4.3 Structural model testing

Based on the normal distribution of sample data, parameter fitting tests were performed on the initial structural model using the maximum likelihood estimation method through AMOS 23.0. The following content provides the results for model fit: *χ*^2^/df = 2.103, below the critical standard of 3; and Root Mean Square Error of Approximation (RMSEA) = 0.043, which satisfied the standard of less than 0.08. The remaining structural equation model fit indices [Goodness of Fit Index (GFI) = 0.946, Adjusted Goodness of Fit Index (AGFI) = 0.931, Normed Fit Index (NFI) = 0.946, Incremental Fit Index (IFI) = 0.981, Comparative Fit Index (CFI) = 0.981, and Tucker-Lewis Index (TLI) = 0.978] met the criterion of >0.9 (Hu and Bentler, 1999), confirming that the model had an acceptable explanatory power and excellent model fit. Therefore, the sample data could be used for subsequent hypothesis testing.

### 4.4 Direct effect test

**Figure 3:**
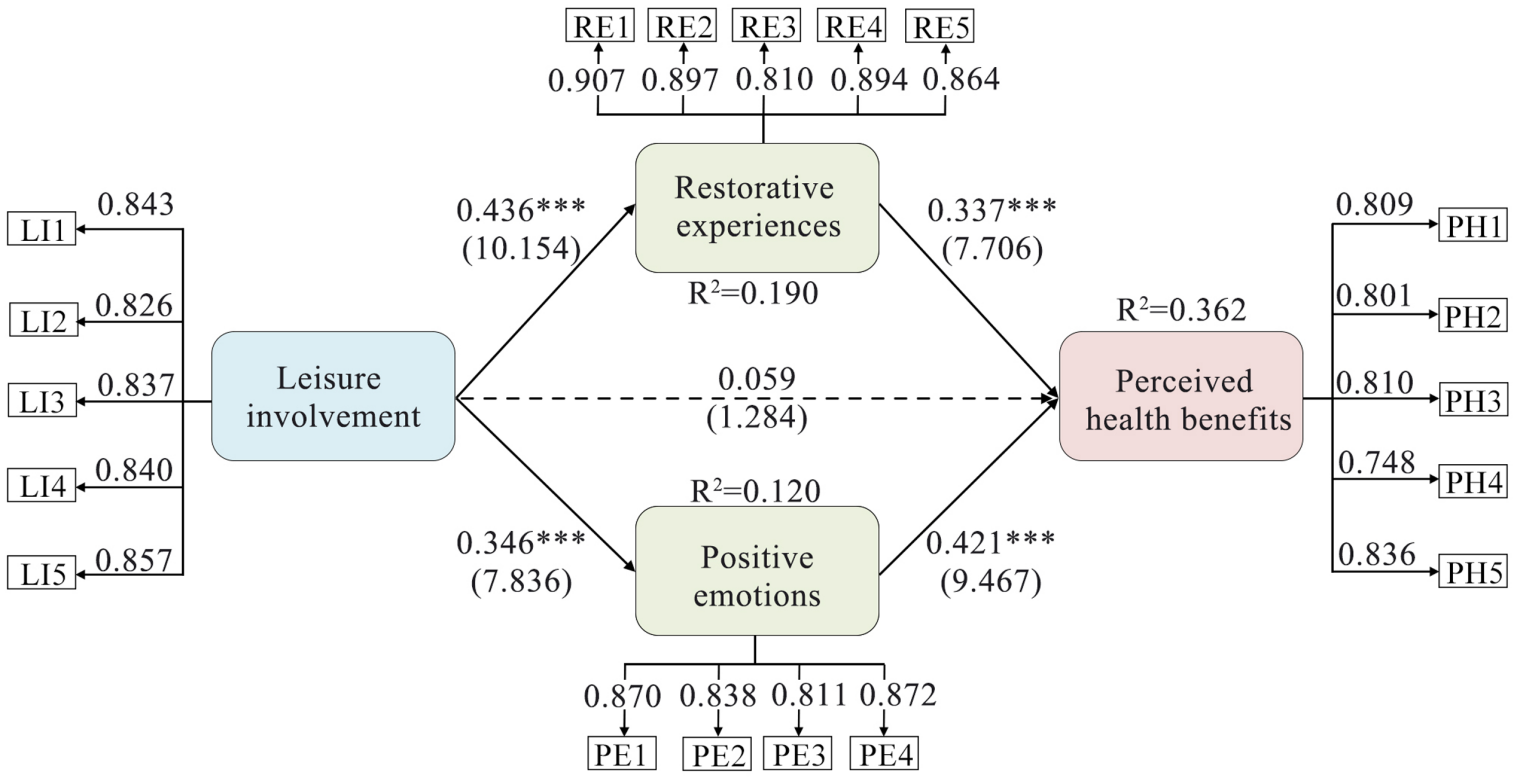
Estimates of structural equation modeling. Dotted line: nonsignificant path; solid line: significant path, standardized coefficient (*t* value); ***p < 0.001.

As shown in Figure 3, leisure involvement significantly positively affected restorative experiences (*β* = 0.436, *p* < 0.001) and positive emotions (*β* = 0.346, *p* < 0.001). Furthermore, both restorative experiences (*β* = 0.337, *p* < 0.001) and positive emotions (*β* = 0.412, *p* < 0.001) positively affected perceived health benefits; thus H2, H3, H5, and H6 were supported. However, the relationship between leisure involvement and perceived health benefits was not significant (*β* = 0.059, *p* = 0.199), leading to the rejection of H1 (Table 5).

**Table 5:** Estimates of direct and mediating effects.

Path	Standardized estimate	95% bias-corrected confidence interval	Hypothesis
Direct effects			
H1: Leisure involvement → Perceived health benefits	0.059	(−0.062 to 0.149)	Unsupported
H2: Leisure involvement → Restorative experiences	0.436***	(0.320–0.536)	Supported
H3: Restorative experiences → Perceived health benefits	0.337**	(0.219–0.463)	Supported
H5: Leisure involvement → Positive emotions	0346**	(0.242–0.457)	Supported
H6: Positive emotions → Perceived health benefits	0.421***	(0.293–0.534)	Supported
Mediating effects			
H4: Leisure involvement → Restorative experiences → Perceived health benefits	0.147***	(0.090–0.221)	Supported
H7: Leisure involvement → Positive emotions → Perceived health benefits	0.145***	(0.093–0.211)	Supported

*p < 0.05; **p < 0.01; and ***p < 0.001.

### 4.5 Mediating effect test

The significance of the mediating effects in this study was confirmed when the 95% bias-corrected confidence interval (CI) did not include zero. Table 5 shows that leisure involvement indirectly affected perceived health benefits through the fully mediating effects of restorative experiences (*β* = 0.147, *p* < 0.001) and positive emotions (*β* = 0.145, *p* < 0.001), thereby supporting H4 and H7.

### 4.6 Moderating effect test

The PROCESS macro (Model 14) of the SPSS software was employed to assess the moderating effects of place attachment. This analysis allowed for a detailed examination of how place dependence and place identity influenced the strength of the mediation paths previously identified in this study.

#### 4.6.1 Moderating effects of place dependence

**Figure 4:**
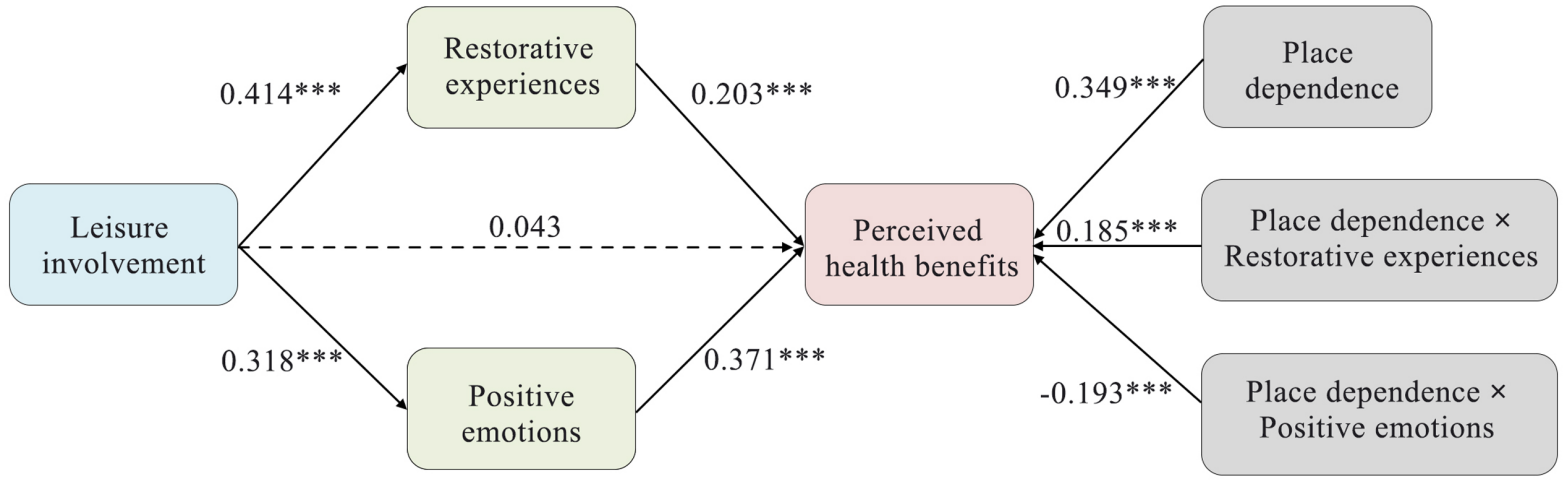
Results of the moderating effect test of place dependence. Dotted line: non-significant path; solid line: significant path; ***p < 0.001.

**Figure 5:**
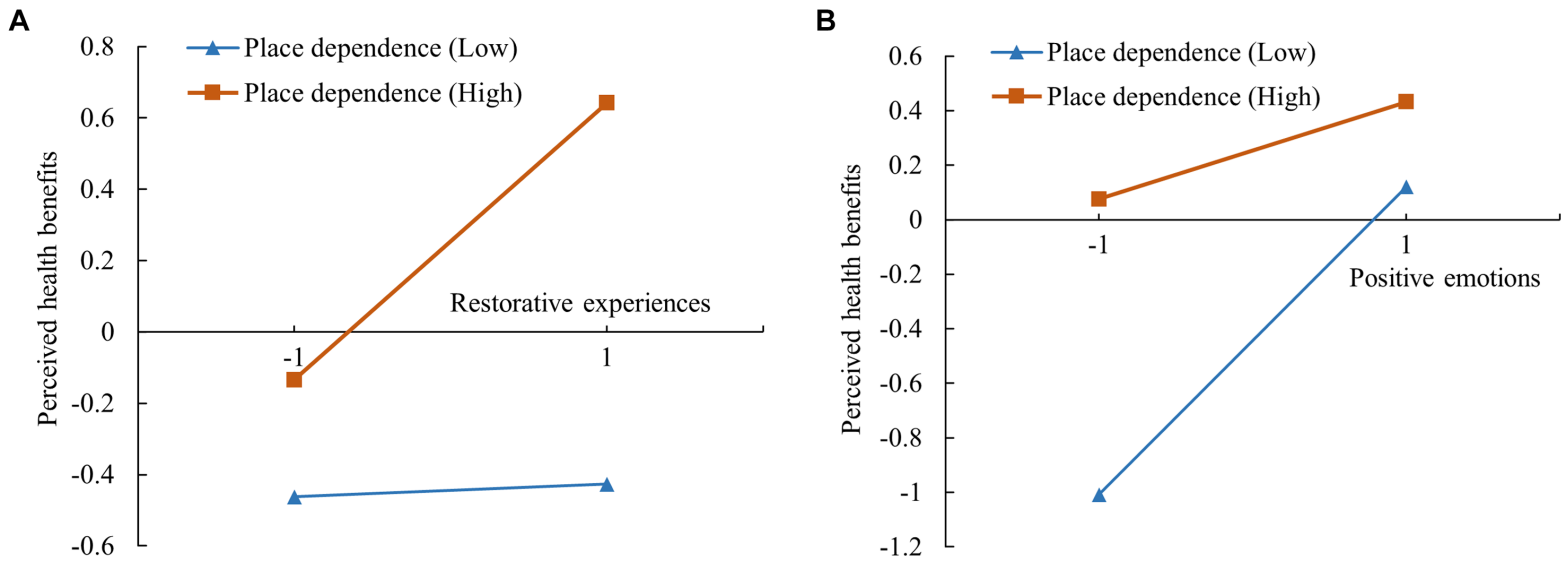
Simple slopes plot of the moderating effect of place dependence: **(A)** Relationship between restorative experiences and perceived health benefits; **(B)** Relationship between positive emotions and perceived health benefits.

Figure 4 shows that the interaction term of restorative experiences and place dependence had a significant positive effect on perceived health benefits (*β* = 0.185, *p* < 0.001). The corresponding moderating effect plot, as shown in Figure 5A, illustrated that the effect of restorative experiences on perceived health benefits was more pronounced for visitors with a high level of place dependence compared with those with low levels. In addition, the interaction term between positive emotions and place dependence had a significant negative effect on perceived health benefits (*β* = −0.193, *p* < 0.001). Figure 5B shows that the effect of positive emotions on perceived health benefits was more pronounced for visitors with low levels of place dependence than for those with high levels.

The test results for mediating effects under the moderation of place dependence are presented in Table 6. The analysis showed that place dependence significantly moderated the mediating effects of restorative experiences (*β* = 0.077, 95% CI = 0.051–0.108) and positive emotions (*β* = −0.061, 95% CI = −0.088 to −0.038).

**Table 6:** Results of moderated mediation effect test.

Moderated mediating pathways	Moderator variable	Indirect effect	Bootstrap 95% confidence interval
Leisure involvement → Restorative experiences × Place dependence → Perceived health benefits	High	0.161	(0.101–0.235)
	Low	0.007	(−0.039 to 0.053)
	Index	0.077	(0.051–0.108)
Leisure involvement → Positive emotions × Place dependence → Perceived health benefits	High	0.057	(0.022–0.097)
	Low	0.179	(0.118–0.244)
	Index	−0.061	(−0.088 to −0.038)
Leisure involvement → Restorative experiences × Place identity → Perceived health benefits	High	0.119	(0.071–0.175)
	Low	0.005	(−0.038 to 0.048)
	Index	0.057	(0.035–0.082)
Leisure involvement → Positive emotions × Place identity → Perceived health benefits	High	0.072	(0.036–0.117)
	Low	0.189	(0.125–0.258)
	Index	−0.058	(−0.087 to −0.035)

#### 4.6.2 Moderating effects of place identity

**Figure 6:**
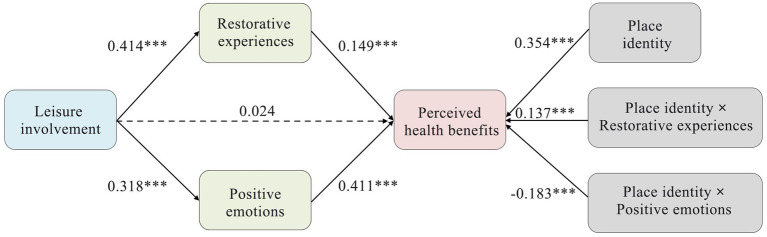
Results of the moderating effect test of place identity. Dotted line: non-significant path; solid line: significant path; ***p < 0.001.

**Figure 7:**
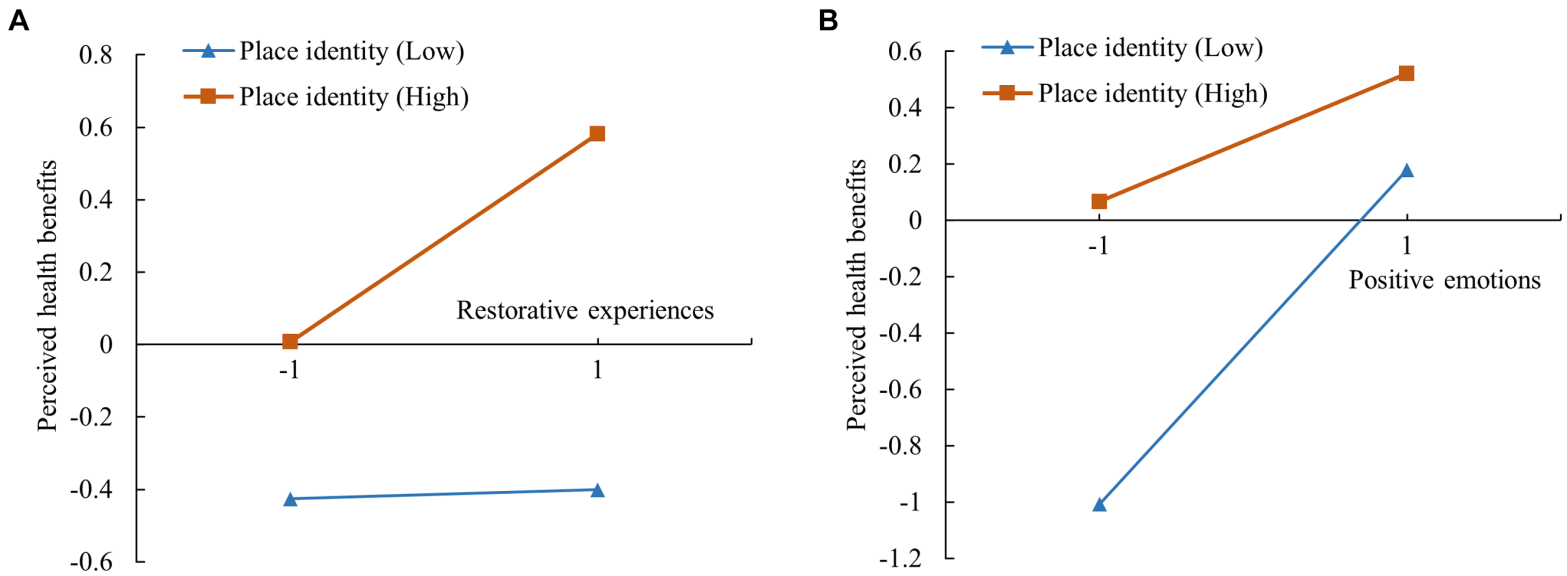
Simple slopes plot of the moderating effect of place identity. **(A)** Relationship between restorative experiences and perceived health benefits; **(B)** Relationship between positive emotions and perceived health benefits.

Figure 6 shows that the interaction term between restorative experiences and place identity had a significant positive effect on the perceived health benefits (*β* = 0.137, *p* < 0.001). The moderating effect plot, as shown in Figure 7A, illustrates that the effect of restorative experiences on perceived health benefits was more pronounced for visitors with a high level of place identity than for those with low levels. In addition, the interaction term between positive emotions and place identity had a significant negative effect on perceived health benefits (*β* = −0.183, *p* < 0.001). Figure 7B shows that the effect of positive emotions on perceived health benefits was more pronounced for visitors with low levels of place identity than for those with high levels.

Table 6 shows that place identity significantly moderated the mediating effects of restorative experiences (*β* = 0.057, 95% CI = 0.035–0.082) and positive emotions (*β* = −0.058, 95% CI = −0.087 to −0.035) in the indirect relationship between leisure participation and perceived health benefits. Therefore, H8 was supported, and H9 was rejected.

## 5 Discussion and conclusion

This study, based on the SORM integrated model, revealed that leisure involvement significantly improved restorative experiences and positive emotions, both of which positively affected perceived health benefits. Despite the insignificant direct relationship between leisure involvement and perceived health benefits, both restorative experiences and positive emotions were found to mediate this relationship.

In addition, place attachment significantly moderated the indirect relationship between leisure involvement and perceived health benefits, underscoring its role as a psychological moderator within the SORM model. Specifically, place attachment positively moderated the direct relationship between restorative experiences and perceived health benefits, thereby positively moderating the mediating effect of restorative experiences. Place attachment negatively moderated the direct relationship between positive emotions and perceived health benefits, thereby negatively moderating the mediating effect of positive emotions.

### 5.1 Discussion

First, this study found that the direct effect of leisure involvement on perceived health benefits was not significant. This result differed from the findings of Geng et al. (2023) that leisure involvement significantly and positively impacted the physiological, psychological, and social health benefits among cyclists. One possible reason was that we measured perceived health benefits as a second-order concept, which was more comprehensive. In addition, differences exist in the strength and specific pathways of influence of different types of landscape settings interacting with health and well-being outcomes (Pietilä et al., 2015; Kong et al., 2022; Tsao et al., 2022). For example, people often do not regularly visit remote scenic areas for leisure activities, yet such settings might be more likely to promote noticeable improvements in physical and mental health and overall well-being due to their distinct separation from urban stressors (Lehto, 2013). In contrast, urban forest parks, while accessible, might require visitors to accumulate more positive mental resources to achieve similar levels of health restoration.

Second, we detected that leisure involvement positively influenced restorative experiences and positive emotions. This finding was consistent with several other studies emphasizing the mental health benefits of nature exposure (Chen H.-T. et al., 2018; Yu et al., 2018). When visitors are highly involved in leisure activities, they become more focused and engaged, enabling them to disconnect from daily stress and fully immerse themselves in the natural environment. This deep involvement not only promotes mental restoration but also enhances their positive emotional experiences (Li et al., 2023). Collectively, these findings reinforced the therapeutic value of urban forest parks, reflecting the growing demand for such leisure spaces among urban residents (Chen B. et al., 2018), not only for relaxation and stress relief, but also as an important component of a healthier lifestyle.

Third, this study found that restorative experiences and positive emotions positively influenced perceived health benefits, thereby validating their roles as full mediators in the relationship between leisure involvement and perceived health benefits. These mediators highlighted the crucial role of psychological states in converting leisure activities into health benefits. The broaden-and-build theory supports this, suggesting that positive emotions foster a deeper subjective engagement with the environment, enhancing restorative experiences and accruing psychological energy (Fredrickson, 2001). This energy then translates into actions that benefit physical and mental health (Pietilä et al., 2015; Chun et al., 2023). For example, Lee et al. (2022) demonstrated the importance of perceived restorativeness and emotional state in measuring health promotion outcomes among tourists, while Gao et al. (2019) found that urban green spaces could trigger various stress-recovery activities, thereby boosting participants’ health levels. Overall, these similar findings confirmed that the acquisition of enhanced psychological resources (i.e., positive emotions and restorative experiences) was necessary to improve the perceived health benefits for visitors.

Fourth, this study confirmed that place attachment not only enhanced the effect of restorative experiences on perceived health benefits but also positively moderated the mediating effect of restorative experiences. Williams and Vaske (2003) noted that people develop place attachment through experience and memory and associate the self with place. People at favorite places typically had higher natural connectedness and positive restorative experiences (Korpela et al., 2008). Similarly, it has been found that natural connectedness as both a mediator and a moderator in developing mental health benefits (Liu et al., 2022). Additionally, this study also supported the results of Liu et al. (2020), who found that place attachment increased tourists’ environmental preference and ultimately affected mental health recovery. A potential explanation for these dynamics was that place attachment fostered a sense of “ownership” among visitors, increasing their willingness to engage in leisure activities. This engagement promoted both a physical and mental “escape,” aiding in recovering psychological resources and further enhancing the perceived health benefits for visitors. Furthermore, place dependence, as a dimension of place attachment, positively moderated the mediating effect of the restorative experiences more strongly, which might be related to the characteristics of visitors. The frequent visits and the stronger material dependence on the leisure environment, which are common in urban forest parks, likely contributed to this effect (Chen B. et al., 2018).

Fifth, this study revealed that place attachment could weaken the impact of positive emotions on perceived health benefits, negatively moderating the mediating effect of positive emotions. This finding deviated from the positive effects of place attachment on mental health and subjective well-being (Wang et al., 2022; Guo et al., 2024). Also, it emphasized the boundary conditions that triggered the mediating effects of positive emotions in the indirect relationship between leisure involvement and perceived health benefits. Research indicated that tourists with high levels of place attachment developed deep emotional connections, focusing more on the health benefits derived from leisure and recreation (Liu et al., 2020). Conversely, those with lower levels of place attachment often exhibited a stronger inclination to explore leisure environments. These visitors were more likely to invest time and energy in enhancing their experiences, seeking and expecting to gain positive emotional experiences from these engagements. Additionally, aesthetic fatigue could diminish interest and positive emotions toward the environment in urban green spaces where landscapes are homogenous (Yan et al., 2024), thus limiting the positive moderating effects of place attachment. This study confirmed that the psychological processes leading to perceived health benefits varied significantly among visitors with different levels of place attachment. Restorative experiences played a dominant role in forming perceived health benefits for frequent visitors to urban forest parks, who generally exhibited high levels of place attachment. In contrast, the role of positive emotions became more critical for visitors with lower levels of place attachment.

### 5.2 Theoretical implications

By focusing on the critical role of visitors’ internal psychological states (Hobfoll, 1989; van den Berg et al., 2003), this study divided the process of perceived health benefits in urban forest parks into three distinct stages: environmental stimulus (leisure involvement) → psychological state (restorative experiences and positive emotions) → psychological response (perceived health benefits). This comprehensive and sequential psychological model uncovered the “black box” relationship between leisure involvement and perceived health benefits, significantly enriching the existing literature.

Additionally, this study innovatively introduced place attachment as a psychological moderator within the SORM model, thereby expanding and advancing the traditional SOR framework and offering new perspectives for future research. Traditionally, place attachment has primarily been viewed as an antecedent or a mediating variable (Liu et al., 2020; Zhou et al., 2023). However, its potential as a moderating variable has been less explored despite suggestions that emotional connections between people and environments significantly shape psychological states and behavioral responses (Kim et al., 2017; Guo et al., 2024). Therefore, this study addressed a significant gap in the literature by emphasizing the moderating role of place attachment in the mechanism influencing perceived health benefits, thus broadening its application in leisure research.

Notably, the study also highlighted the nuanced role of place attachment, demonstrating its dual function as both a positive moderator of the relationship between restorative experiences and perceived health benefits, and a negative moderator between positive emotions and perceived health benefits. These findings not only reveal the boundary conditions under which restorative experiences and positive emotions, as mediating variables, significantly impact health outcomes but also provide valuable insights for developing more targeted and effective health promotion strategies, thereby maximizing the health benefits of natural environments for different groups.

### 5.3 Practical implications

The findings of this study contribute to the development of landscape management strategies in urban forest parks to maximize visitors’ perceived health benefits. First, urban forest parks should cater to diverse leisure needs and actively promote these opportunities through various channels, such as official websites and television advertisements, to boost visitors’ leisure involvement. Parks can enhance their appeal by highlighting the health benefits of the forest environment, and encouraging more local residents to participate in leisure activities for stress relief and emotional well-being. Furthermore, the landscape design should be visitor-centric, incorporating ample leisure facilities and public resources to meet activity preferences and improve the overall leisure experience.

Second, urban forest parks should prioritize the enhancement of the restorative potential of the environments for visitors with high levels of place attachment. This can be achieved by enhancing landscape features such as green visibility, naturalness, and biodiversity, which strengthen sensory interactions with nature and facilitate psychological recovery. Additionally, rational visitor mobility planning can improve the connectivity of the park’s internal and external spaces, thus inducing visitors to actively engage in physical activities (e.g., walking, jogging, etc.) and ultimately achieve physiological recovery.

Third, urban forest parks should focus on fostering positive emotional experiences during leisure activities to enhance the perceived health benefits for visitors with lower levels of place attachment. Recognizing that different age groups have varied psychological needs and preferences—such as excitement and novelty for younger visitors versus comfort and safety for older ones—parks should implement varied leisure routes and activity programs that accommodate different levels of physical exertion and enhance visitor engagement. For instance, introducing themed areas such as forest adventure zones, yoga zones, and quiet meditation areas can cater to diverse interests and enhance visitor satisfaction. Moreover, providing comprehensive humanistic care throughout the leisure process, such as information desks and volunteer services across various activity zones, can improve visitor support and ensure a positive and fulfilling park experience for all visitors.

### 5.4 Limitations and future research

This study had several limitations. First, this study only selected Fuzhou National Forest Park as the study site to explore the influence mechanism of leisure involvement on perceived health benefits. Future studies should test the applicability of the mechanism to other leisure destinations with different levels of development and resource types. Second, perceived health benefits were measured only through questionnaire items in this study. Future research should incorporate physiological measures such as electromyography and heart rate variability to obtain a more comprehensive and objective evaluation of the medical value of urban forest parks in physical and mental health. Third, the use of convenience sampling in this study may limit the generalizability of the findings. Future studies should consider using random sampling methods to enhance the representativeness of the results. Fourth, the process of achieving perceived health benefits is complex and influenced by different variables, including both internal traits and external environmental factors. Future research should include additional potential mediators and moderators, such as age and sex. Finally, visitors engaging in different types of leisure activities may have various pathways to achieving perceived health benefits. Future studies should employ multi-group analyses to explore the differences in the structural models among tourists engaging in diverse leisure activities.

## Data Availability

The raw data supporting the conclusions of this article will be made available by the authors, without undue reservation.
